# A Preliminary Investigation of User Perception and Behavioral Intention for Different Review Types: Customers and Designers Perspective

**DOI:** 10.1155/2014/872929

**Published:** 2014-02-23

**Authors:** Atika Qazi, Ram Gopal Raj, Muhammad Tahir, Mahwish Waheed, Saif Ur Rehman Khan, Ajith Abraham

**Affiliations:** ^1^Faculty of Computer Science and Information Technology, University of Malaya, 50603 Kuala Lumpur, Malaysia; ^2^Faculty of Information Science and Technology, COMSATS Institute of Information Technology (CIIT), Park Road, Islamabad 44000, Pakistan; ^3^Faculty of Computing and Information Technology, King Abdulaziz University, North Jeddah Branch, Jeddah 21589, Saudi Arabia; ^4^Machine Intelligence Research Labs, Scientific Network for Innovation and Research Excellence, Auburn, WA 98071, USA

## Abstract

Existing opinion mining studies have focused on and explored only two types of reviews, that is, regular and comparative. There is a visible gap in determining the useful review types from customers and designers perspective. Based on Technology Acceptance Model (TAM) and statistical measures we examine users' perception about different review types and its effects in terms of behavioral intention towards using online review system. By using sample of users (*N* = 400) and designers (*N* = 106), current research work studies three review types, A (regular), B (comparative), and C (suggestive), which are related to perceived usefulness, perceived ease of use, and behavioral intention. The study reveals that positive perception of the use of suggestive reviews improves users' decision making in business intelligence. The results also depict that type C (suggestive reviews) could be considered a new useful review type in addition to other types, A and B.

## 1. Introduction

Manufacturers, designers, and retailers have long worked to identify the current and future needs of the customers. For this, the industry collects the relevant consumer related data via surveys and other types of research studies. However, the opinion of customers is also significantly reflected in the form of online reviews which help prospective consumers in their purchase decision making [1, 2]. Based on these reviews, manufacturers, product designers, and retailers can predict the needs and preferences of the customers. They, in turn, make their products and services more customer focused and improve sales figure. The product designers may get useful information from these online reviews in order to better understand the voice of customers. There are such situations where users have few opinions and recommendations cannot be made properly, therefore to address this issue the advanced algorithm is proposed in [3].

These reviews are differentiated on the basis of language constructs that express different types of information [1]. The research so far has identified only two types of reviews (or opinions), that is, regular and comparative [4]. There is a visible gap in determining further useful review types in the perspective of designers and customers. This provides a valuable space to uncover more useful review types and extend users' perception. The users are the main entity of online review system that exchanges the information with each other in the form of reviews. Therefore, users' acceptance to use online review system and share their valuable opinions is highly required. To determine this individual acceptance, the perceived ease of use (PEOU) and perceived usefulness (PU) have been considered the important factors to measure [2]. These factors could probably be best tested on the valuable data obtained from different aspects of WWW such as education, commerce, trade, showbiz, and interpersonal flow of information.

Technology Acceptance Model (TAM) [5] could be helpful in examining these factors as a function of users' perception about a system. Existing work [6] has mostly considered the aspects of user and system characteristics using TAM. This study, however, deals with the aspects of (1) different review types as constructs (type A, type B, and type C) using TAM in order to analyze users' perception towards using online review system and (2) classifying the review types by manual labeling as described in previous studies of regular and comparative reviews [7]. This study also focuses on an extensive quantitative analysis of survey data by employing different statistical methods such as regression, correlation and mediation analysis in order to determine the users' perception and behavioral intention and to establish the authenticity of our work.

The rest of this paper is organized as follows.  Section 2 introduces the concepts and related work. In Section 3, we present the research model and hypotheses. In Sections 4 and 5, we describe our research design and results. Finally, Section 6 provides discussion, limitation, and the future work.

## 2. Literature Review

### 2.1. Opinion Types

Web 2.0 acts as an interactive platform for users to share their views, sentiments, and opinions as reviews (postings). These posted data (or opinions) are generally of two types, subjective and objective, but mostly include the subjective expressions [8, 9]. Based on language constructs, the opinionated data can describe different types of information [1] and can further be classified into two types, that is, regular opinions and comparative opinions [4]. A regular opinion describes a single entity while a comparative opinion deals with two or more entities. For example, a regular opinion is mostly used to find good or bad views about a particular product whereas a comparative opinion is significantly utilized for comparing two or more products (or simply to describe the competitive intelligence involved in these products). Existing work [4, 10–12] covers different aspects of regular opinions.

The concept of comparative sentence mining originates from the inspiring work of [1] and is then explored further by other researchers [8, 13–15]. Jindal and Liu [1] proposed an efficient scheme which identifies the comparative sentences by using selected datasets. Hou and Li [13] proposed a technique for Chinese comparative sentence mining.

Furthermore, Xu et al. [14] proposed a technique to identify product strengths and weaknesses by looking into comparative sentences. Xu et al. [15] provided a method of comparative relation extraction that not only detects the occurrence of relations but also recognizes direction (positivity or negativity) of reviews.

The views or opinions are analyzed using different opinion mining (OM) methods. This analysis employs various techniques from different domains such as natural language processing, computational linguistics, and text mining [16]. All discussed studies above describe the evaluation of comparative and regular reviews. In order to identify more useful review types from customers and designers perspective, we use TAM to determine significant review types by analyzing users' perception and behavioral intention in using online review system.

### 2.2. Technology Acceptance Model (TAM)

The Technology Acceptance Model (TAM) [2] is considered a powerful model for system acceptance and users' usage behavior [17]. TAM is the successor of Theory of Reasoned Action (TRA) [18] which is used to explain and predict the acceptance of technology across the diverse user groups and information systems. TAM proposes measures for users' acceptance of an information system such as perception of the ease of use that is, PEOU (the measure of users' belief that using a particular system would be easy to use), perceived usefulness, that is, PU (it is the measure of users' belief that using a particular system would improve their performance towards the job), behavioral intention, that is, BI (it is a measure of users' responsiveness for liking/disliking to use a particular system in the future), and actual use, that is, AU of the system which determines the measure of actual performance in comparison to expected performance of a particular system.

TAM has widely been used as the basis of the research that aims to examine the behavior of the users as well as the usage intentions [5, 19]. A diverse range of the computing techniques, user populations, and organizational settings have been successfully tested using TAM. For instance, usage of graphic systems by the school teachers [20] and students acceptance of the applications such as Microsoft Word, Excel, PowerPoint, and Access [21] is successfully examined by TAM. It has also been used to find the user's perception about text editor applications and the electronic mail systems [23]. Similarly, TAM has been used in numerous studies in other different domains, for example, a telemedicine technique [20] in health care, online consumer as buyers behavior [24], the web based survey tools [17], the understanding of the interface styles [25], the communication attributes of users towards the computer-based environment [26], the modification of innovative techniques for the experienced as well as the new users [27], and the users' acceptance of computerized communication media [28]. The prior research efforts using TAM are based on the constructs such as usefulness, ease of use, and on the factors that influence users' acceptance of informative systems [29]. As indicated in [19] the perception of these structures helps and facilitates the acceptance of innovative information systems. An online reviews system provides users with a platform to interact with one another via reviews. The reviews are the basic parts of the online review systems and, therefore, act as strong attractive factors for decision making. The user's attitude towards the acceptance or rejection of a review (and the categorization into different customized review types) is determined by different factors including the context of review, its usefulness in that context, the ease of adaptation, and the related variables that fit in the contexts of future user tasks.

## 3. Research Model and Hypotheses

**Figure 1:**
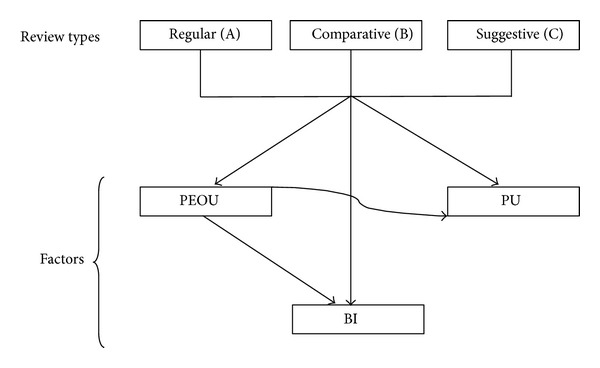
The proposed model.

The proposed research model is presented in Figure 1. The model is based on TAM and on previous studies [30, 31]. The model describes that adoptability of multiple review types available by means of information systems (blogs, discussion forums, and ecommerce and healthcare sites, etc.) plays an important role for the success in decision making. The measurable variables such as perceived ease of use (PEOU), perceived usefulness (PU), and behavioral intention (BI) are used to find the users' interest towards using opinions for improving their decision making. These factors have been evaluated in the context of customers and designers while considering the TAM in perspective (see results in Section 5).

Users interact in an online review system by sharing a variety of reviews on various topics, products, and so forth. The success level of a newly launched product increases based on the relative intensity of customer approval (opinion) [32], that is, the more the review is helpful the more the user satisfaction increases. This argument strongly supports the assumption that review types are positively related to perceived usefulness (as is suggested in the model above). The review system that allows users to classify variety of reviews on the basis of certain factors (e.g., PEOU and PU) is beneficial for users for decision making and thus enhances the ease of use [4, 33].

These reviews have different types, where each type has its own features. The review types grab users' interest by providing solutions and enhance the decision-making skills. These review types provide greater encouraging assessments from users than review types which do not have such facilities or features (or more specifically which are general). Keeping in view the concepts of users' decision making and organizational change, user participation, user acceptance, behavioral intention, usage, and satisfaction with the system we will focus on the relationship between users' perception on review types and behavioral intention. We will examine the meditating factor among these relationships through mediation analysis that is supported by TAM. Mediating relationships occur when a third variable such as PU/PEOU plays a leading role between independent (A, B, and C) and dependent variables (BI). Thus, in the context of the above discussion, the findings of existing studies on TAM, and the review types, the researchers propose the hypotheses given in Table 1.

All our propositions are supported by findings in literature; that is, they are consistent with TAM and its related research studies [2, 22, 28].

## 4. Research Design

In the pilot study, we collected and analyzed 250 reviews from Amazon, 200 reviews from blogs, and 400 reviews from a self-deployed website (http://www.reviewscollection.com/) exclusively developed for this study (total of 506 users including 400 customers and 106 designers participated in the study). We assigned each review to a specific category based on its inherent meanings (semantics). The degree of prediction (so that a review belongs to a certain category) was measured by manually assigning the labels: A, B, and C for *regular reviews*, *comparative reviews*, and *suggestive reviews*, respectively. This pilot study method resembles the closed card sorting method [34]. By using linguistics types A and B are identified by the authors [1]. We have used same approach to find more useful types and identified *suggestive* as a third innovative review type. Suggestive reviews are the speech acts which are used to direct someone to do something in the form of a suggestion. The review types are used as external constructs with TAM.

Four items, each from the well-defined scales [19], were used to measure perceived ease of use and perceived usefulness. The reliability of perceived usefulness for customer is .70 and for perceived ease of use is .79 (Table 3), whereas the reliability for designer data of perceived usefulness is .71 and perceived ease of use is .83 (Table 4). We use three items from the study [19] and four items from [35] to measure behavioral intention. The reliability for customer is .71 and for designer is .70 (Tables 3 and 4). Also the scale items for review types A, B, and C are based on the results of previous studies [12, 36–39] in which each study focuses on the maximum benefits in terms of decision making, problem solving, business intelligence, and policy making by considering reviews reading and writing. The cronbach alpha reliability for customers on reviews type A is .80, for B is .80, and for C is .70, whereas cronbach alpha reliability for designers' data for reviews type A is .76, for B is .72, and for C is .70 (Tables 3 and 4). We have used factor analysis for customers' and designers' data for the proposed types of reviews A, B, and C (Table 2). A larger scale examination was then conducted with the help of a questionnaire to ascertain the compatibility of the proposed model. All the survey items are measured on 5-point Likert scale ranging from 1 (strongly agree) to 5 (strongly disagree).

## 5. Results

We have presented our results for both users, that is, customers and designers. First we will discuss the results for customers. Regression analysis was used to test the sets of hypotheses H1–H9. We then studied the correlations among the variables which are presented in Table 5.

The correlation results support all our hypotheses. Table 5 reveals that all the correlations are <.80. This indicates that the study variables do not contain multicollinearity [40]. The reliabilities of all review types (A, B, and C) and TAM (BI, PEOU, and PU) are above .70. To confirm these hypotheses we used regression analysis, that is, commonly used to analyze the relationship between dependent and independent variables, for example, as in our study, the review types (independent variables) and PEOU, PU, and BI (dependent variables). The results of the regression analysis for customers' data are presented in Table 6.

Result in Table 7 reveals that PEOU does not mediate between type B and behavioral intention for hypothesis 11, whereas hypothesis 12 predicts the mediating affect of PEOU between type C and behavioral intention. Results suggest considerable decrease in Beta value of type C (from *β* = .60, *P* = .000, to *β* = .13, n.s.) which shows that PEOU fully mediates between type C and behavioral intention. Hypothesis 13 predicts that PU mediates between review type A and behavioral intention. Result reveals that PU does not mediate between the relationship between type A and behavioral intention, whereas hypothesis 14 predicts the mediating effect of PU between type B and behavioral intention. Results suggest considerable decrease in Beta value of type B (from *β* = .37, *P* = .000, to *β* = .26, *P* = .000) which shows that PU partially mediates between type B and behavioral intention. Hypothesis 15 predicts the mediating effects of PU between type C and behavioral intention. Results suggest considerable decrease in Beta value of type C (from *β* = .23, *P* = .000 to *β* = .04, n.s.) which shows that PU fully mediates between type C and behavioral intention. The same tests are repeated for designers' data and the results are provided in Table 8.

The reliabilities of all review types (A, B, and C) and TAM (BI, PEOU, and PU) are above .70. The correlation results support our all hypotheses except H1, H4, H7 and H9. To confirm these hypotheses we used regression analysis. The results of the regression analysis are presented in Table 9.

As Table 9 shows, review type A had no significant effect on perceived ease of use (*β* = .02, n.s.), review type B had significant effect on perceived ease of use (*β* = .70, *P* = .000), and review type C had significant effect on perceived ease of use (*β* = .19, *P* = .05). Review type A did not have significant effect on perceived usefulness (*β* = .06, N.S), review type B had significant effect on perceived usefulness (*β* = .24, *P* = .011), and review type C had significant effect on perceived usefulness (*β* = .70, *P* = .000). The direct effect of review type A on behavioral intention had no significant effect (*β* = .11, N.S), whereas review types B (*β* = .60, *P* = .000) and C (*β* = .20, *P* = .040) show their significance. The perceived ease of use had significant effect on BI (*β* = .50, *P* = .000) and perceived usefulness had also significant effect on behavioral intention (*β* = .25, *P* = .010). We did not check hypotheses 10 and 13; the reason behind it is that type A does not fulfill the condition prescribed by [41]. Result of mediation analysis shown is in Table 10.

Results reveal that for hypothesis 11 PEOU partially mediates between review type B and behavioral intention (from *β* = .50, *P* = .000, to *β* = .3, *P* = .000). Hypothesis 12 predicts that PEOU fully mediates between type C and BI (from *β* = .25, *P* = .0, to *β* = .13, n.s.). Hypothesis 14 predicts that PU mediates between review type B and BI (from *β* = .60, *P* = .000, to *β* = .26, *P* = .000) which shows that PU partially mediates between type B and behavioral intention. Hypothesis 15 predicts the mediating effects of PU between type C and behavioral intention. Results suggest considerable decrease in Beta value of type C (from *β* = .20, *P* = .04, to *β* = .001, n.s.) which shows that PU fully mediates between type C and behavioral intention.

## 6. Discussion and Conclusion

In this research work we observed the effect of review types on users' perception and intention by considering the significant effects on different factors, that is, perceived ease of use, perceived usefulness, and behavioral intention. Results indicate that there exists a reasonably good support for many hypotheses presented. In particular, eight of the nine hypotheses concerning review types were confirmed for customers' data. Similarly for mediation analysis four out of five hypotheses are confirmed. For designers' data, four out of five hypotheses are confirmed while for mediation analysis four out of six hypotheses are confirmed.

The results describe that, for customers' data, perceived usefulness is found to be significantly influenced by all the review types, A, B, and C. Also, perceived usefulness is a strong mediator for review type C and provides a significant direct effect on behavioral intention. For type B, the perceived usefulness partially mediates, while for type C usefulness factor is not supported mediator towards behavioral intention. The results indicate that the comparative reviews enable customers to perceive usefulness by taking better purchasing decisions in business environments which in turn increases their intention towards using information systems [1]. Results also demonstrate that more customers have positive intention towards using a particular product or a system due to the perceived usefulness obtained from review type C (suggestive reviews).

Also the perceived ease of use is found to be significantly influenced by the review types B and C. The mediation results of type B show that perceived ease of use does not mediate between type B and behavioral intention while the perceived ease of use fully mediates for review type C. This indicates the significance of type C (suggestive reviews) in addition to review types A and B (regular and comparative reviews, resp.) for behavioral intention.

Results in designers' perspective indicate that review type A (regular reviews) has no influencing effect on perceived usefulness, perceived ease of use, and behavioral intention, whereas the designers perceived that type B and C significantly influence their perception of usefulness and ease of use which in turn leads to positively influence their behavioral intention towards using a review system. This is in consistence with previous studies that review type B (comparative reviews) is more demanding and beneficial for designers [1]. Review type C (suggestive reviews) can help in making better decision because suggestions are common in everyday life interpersonal communication also known as speech acts [42]. Study [43] indicates that suggestions are indeed beneficial for listeners in any form (textual or verbal). The mediation results show that review type C builds a strong perception of use which motivates designers towards their behavioral intentions in using online review system. Also we can conclude that type A for customers is acceptable but not for designers, whereas types B and C are acceptable for both customers and designers.

Overall analysis shows that type C is more significant for both customers and designers to find more usefulness that ultimately improves their satisfaction level. This is in consistence with the previous study [32] which indicates that the possibility of the success of a new product depends mostly on the associated level of customer satisfaction. Furthermore, focused reviews may increase business processing capabilities due to more useful contents they hold. The results show that type C (suggestive reviews) helps designers in designing products with new features depending on customer's input (actual suggestion). Type C helps customers in voicing required features or recommendations (in the form of suggestive opinions) to the product management teams which could be incorporated in new releases of products.

The results of this study for designers indicate that the effect of perceived ease of use (*β* = .50) on behavioral intention is somewhat stronger than that of perceived usefulness (*β* = .50 > *β* = .25). This is in contrast with the results of customers (*β* = .10 < *β* = .23) on perceived ease of use and perceived usefulness, respectively. It has been observed that PEOU and PU are interrelated and this could possibly depend upon the level of experience and category of participants (customer/designer). Both types of participants have different views of the (new and unfamiliar) system at first use. And, they care about their ability to learn and use the system instead of its inherent usefulness. This result is in accordance with [44] which describes that when a user learns enough to use a system, he/she can further easily explore the functionality and the usefulness of the system.

In this research work, we explored different review types including one new proposed type, that is, suggestive review, while considering the TAM in perspective. This further helped us in measuring certain related factors, that is, PEOU, PU, and BI, and the results are described above in different tables. Our study shows that the review types play a significant role in developing the perception of users about a new product or a system while considering the above factors in context. One limitation of the current study is that it is done in a specific and limited environment and could yield bit different results when performed in a professional environment. As a future work, the researchers are encouraged to extract different review types from different domains, for example, discussion forums, blogs, chatting archives, survey responses, and so forth. If a research study combines few domains the results could even be different. Another important point to consider is that the proposed review type C (suggestive reviews) could be helpful in the usability evaluation of different opinion mining systems involving hybrid aspects of reviews.

A promising research area would integrate different review types and evaluate them in different opinion mining contexts. We would also like to investigate the suggestive reviews while keeping in view the customers' and designers' mental capabilities especially to memory types (short term, long term, and prospective). As designers are key components of a business system and they need useful reviews to enhance decision making and improve business policies to develop new products, for business intelligence, more focused reviews (e.g., suggestive) are needed which can help designers develop new products while keeping in view the customers' satisfaction level which can ultimately lead them to a successful business.

## Figures and Tables

**Table 1:** Research hypotheses.

Variables	Hypotheses
Review types	H1: review type A is positively related to perceived ease of use
	H2: review type B is positively related to perceived ease of use
	H3: review type C is positively related to perceived ease of use
	H4: review type A is positively related to perceived usefulness
	H5: review type B is positively related to perceived usefulness
	H6: review type C is positively related to perceived usefulness
	H7: review types A is directly related to behavioral intention to use online review system
	H8: review type B is directly related to behavioral intention to use online review system
	H9: review type C is directly related to behavioral intention to use online review system

Perceptions of ease of use	*Mediation hypothesis *
	H10: perceived ease of use mediates between review type A and behavioral intention
	H11: perceived ease of use mediates between review type B and behavioral intention
	H12: perceived ease of use mediates between review type C and behavioral intention

Perceptions of usefulness	*Mediation hypothesis *
	H13: perceived usefulness mediates between review type A and behavioral intention
	H14: perceived usefulness mediates between review type B and behavioral intention
	H15: perceived usefulness mediates between review type C and behavioral intention

**Table 2:** Factor analysis for review types.

Factor analysis	Customer	Designer
Factor 1: type A		
Enhanced decision making	.68	.76
Gain knowledge	.61	.62
Avoid problem	.79	.75
Share experience	.83	.51
Prefer to read	.77	.60
Prefer to write	.55	.63
I demand more	.44	.61
Factor 2: type B	—	—
Enhanced decision making	.69	.48
Gain knowledge	.62	.52
Avoid problem	.78	.59
Share experience	.81	.62
Prefer to read	.75	.70
Prefer to write	.56	.67
I demand more	.48	.67
Factor 3: type C	—	—
Enhanced decision making	.63	.72
Gain knowledge	.69	.43
Avoid problem	.76	.70
Share experience	.68	.62
Prefer to read	.53	.55
Prefer to write	.47	.64
I demand more	.51	.49

**Table 3:** Customer reliability and descriptive statistics.

Factor	Mean	Standard deviation	Cronbach *α*	Number of items
Type A	3.258	.784	.80	7
Type B	3.073	.733	.80	7
Type C	3.267	.777	.72	7
BI	3.346	.844	.71	3
PU	3.252	.657	.70	4
PEOU	3.251	.859	.79	4

**Table 4:** Designer reliability and descriptive statistics.

Factor	Mean	Standard deviation	Cronbach *α*	Number of items
Type A	3.177	.614	.76	7
Type B	3.009	.634	.72	7
Type C	2.994	.584	.70	7
BI	2.792	.693	.70	3
PU	3.214	.764	.71	4
PEOU	3.143	.912	.83	4

**Table 5:** Customers' correlation among variables.

Variable	1	2	3	4	5	6
(1) Type A						
(2) Type B	.32**					
(3) Type C	.23**	.46**				
(4) BI	.36**	.36**	.17**			
(5) PU	.17**	.37**	.22**	.23**		
(6) PEOU	.06	.11*	.60**	.11**	.23**	—

All correlations are significant at the 0.01 level. *shows significance and **shows more significant values.

**Table 6:** Regression analysis for customer' data.

Dependent variables	*R* ^2^	Independent variables	*β*	*t*	*P*
Perceived usefulness	.03	A	.16	3.35	.001
	.14	B	.37	7.92	.000
	.05	C	.23	4.65	.000

Perceived ease of use	.01	A	.07	1.30	.192
	.02	B	.11	2.16	.03
	.38	C	.60	15.66	.000

Behavioral intention	.12	A	.36	7.51	.000
	.13	B	.37	7.95	.000
	.03	C	.17	3.40	.001
	.06	PU	.23	4.70	.000
	.02	PEOU	.10	2.14	.03

**Table 7:** Mediation analysis of customers' data.

	Behavioral intention			
	*β*	*R* ^2^	Δ*R* ^2^	*P*
*Main effect *				
Step 1				
Perceived usefulness (PU)	.23	.05		.000
Step 2				
Type A	.268			.000
Type B	.262			.000
Type C	.04	.20	.15	.430
Step 1				
Perceived ease of use (PEOU)	.10	.01		.03
Step 2				
Type A	.27			.000
Type B	.32			.000
Type C	.13	.20	.19	.48

**Table 8:** Designers' correlation among study variables.

Variable	1	2	3	4	5	6
(1) Type A						
(2) Type B	.32**					
(3) Type C	.23**	.46**				
(4) BI	.36**	.36**	.17**			
(5) PU	.17**	.37**	.22**	.23**		
(6) PEOU	.06	.11*	.60**	.11**	.23**	—

All correlations are significant at the 0.01 level. *shows significance and **shows more significant values.

**Table 9:** Regression analysis of designers' data.

Dependent variables	R^2^	Independent variables	*β*	t	P
Perceived usefulness	.01	A	.06	.70	.48
	.06	B	.24	2.60	.011
	.54	C	.70	11.08	.000

Perceived ease of use	.01	A	.02	.20	.83
	.62	B	.75	13.28	.000
	.05	C	.19	1.97	.050

Behavioral intention	.02	A	.11	1.14	.25
	.36	B	.60	7.74	.000
	.04	C	.20	2.06	.040
	.06	PU	.25	2.61	.010
	.31	PEOU	.50	6.92	.000

**Table 10:** Mediation analysis of designers' data.

	Behavioral intention			
	*β*	*R* ^2^	Δ*R* ^2^	*P*
*Main effect *				
Step 1				
Perceived usefulness (PU)	.24	.06		.010
Step 2				
Type B	.57			.000
Type C	.001	.37	.31	.90
Step 1				
Perceived ease of use (PEOU)	.56	.31		.000
Step 2				
Type B	.41			.001
Type C	.06	.38	.07	.380
