# Corrigendum to “A Preliminary Investigation of User Perception and Behavioral Intention for Different Review Types: Customers and Designers Perspective”

**DOI:** 10.1155/2015/258150

**Published:** 2015-04-07

**Authors:** Atika Qazi, Ram Gopal Raj, Muhammad Tahir, Mehwish Waheed, Saif Ur Rehman Khan, Ajith Abraham

**Affiliations:** ^1^Faculty of Computer Science and Information Technology, University of Malaya, 50603 Kuala Lumpur, Malaysia; ^2^Faculty of Information Science and Technology, COMSATS Institute of Information Technology (CIIT), Park Road, Islamabad 44000, Pakistan; ^3^Faculty of Computing and Information Technology, King Abdulaziz University, North Jeddah Branch, Jeddah 21589, Saudi Arabia; ^4^Machine Intelligence Research Labs, Scientific Network for Innovation and Research Excellence, Auburn, WA 98071, USA

The author name Mahwish Waheed should be replaced with Mehwish Waheed.

In the body of the paper,

 (from *β* = .50, *P* = .000, to *β* = .3, *P* = .000) should be replaced with the following values (from *β* = .75, *P* = .000 to *β* = .41, *P* = .001);
 (from *β* = .25, *P* = .0, to *β* = .13, n.s.) should be replaced with the following values (from *β* = .19, *P* = .05 to *β* = .06, n.s.);
 (from *β* = .60, *P* = .000, to *β* = .26, *P* = .000) should be replaced with the following values (from *β* = .24, *P* = .011 to *β* = .57, *P* = 000);
 (from *β* = .20, *P* = .04, to *β* = .001, n.s.) should be replaced with the following values (from *β* = .70, *P* = .000 to *β* = .001, n.s.).

Figure 1 is corrected as follows.

## Figures and Tables

**Figure 1:**
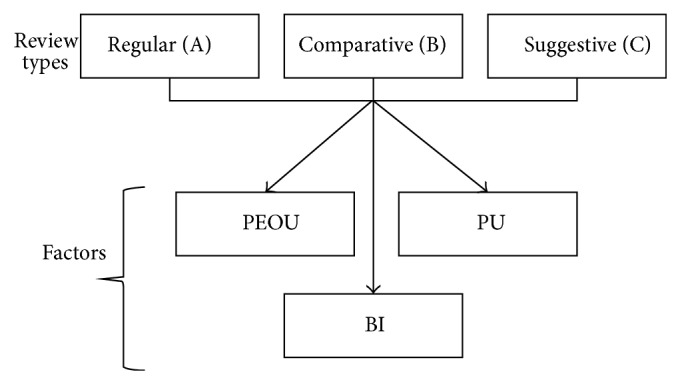
The proposed model.

